# Case Report: The rare pancreatic involvement in Erdheim-Chester disease

**DOI:** 10.3389/fimmu.2025.1611452

**Published:** 2025-07-14

**Authors:** Ji Li, Ming Zhang, Yadan Zou, Jing Zhang, Juanjuan Song, Ting Li, Sheng-Guang Li

**Affiliations:** ^1^ Department of Rheumatology and Immunology, Peking University International Hospital, Beijing, China; ^2^ Department of Pathology, Peking University International Hospital, Beijing, China; ^3^ Department of Nuclear Medicine, Peking University International Hospital, Beijing, China

**Keywords:** Erdheim-Chester disease, pancreatic involvement, BRAF V600E, non-Langerhans histiocytosis, foamy histiocytes, MAPK pathway

## Abstract

**Background:**

Erdheim-Chester disease (ECD) is an exceedingly rare non-Langerhans histiocytosis. While often affecting the skeleton, cardiovascular system, and kidneys, pancreatic involvement remains uncommon and can mimic more prevalent conditions such as autoimmune or chronic pancreatitis.

**Case Presentation:**

A 58-year-old female presented with a two-year history of bilateral lower limb edema and a year-long course of recurrent abdominal pain. Imaging suggested necrotizing pancreatitis and retroperitoneal infiltration, yet serum IgG4 levels were normal. A CT-guided biopsy of the pancreas and retroperitoneum revealed diffuse proliferation of foamy histiocytes (CD68^+^, CD163^+^, CD1α^-^) carrying the BRAF V600E mutation, confirming ECD. Supportive therapy and corticosteroids temporarily relieved symptoms, but targeted treatment was delayed due to the COVID-19 pandemic. Subsequent follow-up revealed significant clinical improvement following targeted therapy.

**Discussion:**

ECD can present with non-specific clinical features, leading to frequent misdiagnoses. Involvement of the pancreas, as demonstrated here, is particularly rare. The discovery of the BRAF V600E mutation underscores the importance of molecular testing for both diagnostic confirmation and therapeutic stratification. The immunopathogenesis of ECD involves activated macrophages and aberrant MAPK signaling, which drive chronic inflammation and tissue fibrosis.

**Conclusion:**

This case highlights the diagnostic challenges of pancreatic ECD and underscores the critical value of an integrated approach—including imaging, immunohistochemistry, and molecular analysis—in achieving timely diagnosis. Early recognition and targeted therapy may significantly improve outcomes for patients with BRAF-mutant ECD.

## Introduction

Erdheim-Chester disease (ECD) is a rare non-Langerhans cell histiocytosis first described by Austrian pathologists Jakob Erdheim and William Chester in 1930 (1). Characterized by the pathological infiltration of lipid-laden (foamy) histiocytes, ECD can affect multiple organ systems—most frequently the long bones, cardiovascular system, and kidneys (2). Although it remains an uncommon diagnosis, advancements in immunohistochemistry and molecular genetics have improved both recognition and understanding of this entity. In particular, the detection of the BRAF V600E mutation in a large proportion of cases has shed light on potential pathogenic mechanisms and therapeutic targets (3).

Despite growing awareness, ECD continues to pose diagnostic and therapeutic challenges. Clinically, patients may present with nonspecific signs and symptoms such as bone pain, malaise, weight loss, and organ dysfunction, making it difficult to distinguish from infections, malignancies, or other inflammatory processes. Imaging modalities—including computed tomography (CT), magnetic resonance imaging (MRI), and positron emission tomography (PET)—often reveal characteristic lesions, such as sclerotic changes in long bones and infiltrative lesions in retroperitoneal or mediastinal spaces (4). However, definitive diagnosis relies on histopathological examination demonstrating foamy histiocytes that are typically CD68- and CD163-positive but CD1a-negative (5). When tissue is available for molecular testing, the presence of the BRAF V600E mutation (or other MAPK pathway mutations) further supports the diagnosis.

Although the skeleton, retroperitoneum, central nervous system, and heart are commonly implicated in ECD, pancreatic involvement remains extremely rare. Only a handful of such cases have been documented in the literature to date (6–9). Consequently, physicians may not routinely consider ECD when evaluating patients with pancreatic mass-like lesions, atypical pancreatitis, or recurrent abdominal pain. Delayed recognition may result in prolonged diagnostic workups, missed opportunities for targeted interventions, and increased morbidity.

In recent years, therapeutic approaches for ECD have evolved beyond high-dose corticosteroids and interferon-α, with BRAF inhibitors and other targeted agents significantly altering prognosis in patients harboring specific mutations (10). Nonetheless, early diagnosis—especially when the pancreas is involved—remains paramount in optimizing outcomes. Here, we present a new case of Erdheim-Chester disease with pancreatic involvement, illustrating how this rare manifestation may mimic more prevalent diseases, including pancreatic malignancy and autoimmune or infectious pancreatitis. We also review the literature on reported cases, highlighting the diagnostic pitfalls, treatment considerations, and overall prognosis of this unusual presentation.

## Case presentation

A 58-year-old woman was admitted to our hospital on November 28, 2022, with a two-year history of progressive bilateral lower limb edema and recurrent abdominal pain for more than one year, worsening notably during the preceding month. Her lower limb edema first appeared in October 2020 without identifiable triggers, and routine urinalysis showed no significant proteinuria at that time, hence no specific treatment was initiated.

Beginning in February 2021, she experienced episodes of intermittent abdominal pain associated occasionally with brief periods without bowel movements or flatus, but she denied nausea or vomiting. Initial laboratory tests showed mild leukocytosis (white blood cell count of 10.69 × 10^9/L), hypoalbuminemia (24.99 g/L), and normal renal function (serum creatinine 51.6 μmol/L). She was managed conservatively for suspected intestinal obstruction with antibiotics and laxatives, achieving transient symptom relief; however, lower limb edema progressively worsened.

Approximately one month later, ultrasonography revealed bilateral hydronephrosis and dilated upper left ureter, prompting suspicion of renal tuberculosis. Empirical anti-tuberculosis treatment (isoniazid, rifampin, ethambutol, and levofloxacin) was commenced. Despite this regimen, after about a month, she developed persistent fever reaching 39°C, which did not respond to antibiotics alone. Elevated inflammatory markers (ESR 29 mm/h, CRP 267.69 mg/L), mild elevation of procalcitonin (0.27 ng/mL), and severe hypoalbuminemia (17.8 g/L) were noted. Chest CT showed bilateral interstitial changes compatible with bronchiolitis and a small pericardial effusion. Her fever resolved promptly after initiating corticosteroids (methylprednisolone 24 mg/day) but returned when corticosteroids were tapered off.

Six months after treatment initiation, her abdominal pain became persistent, presenting as a distending ache radiating to her back, somewhat relieved by leaning forward. Subsequent abdominal CT indicated diffuse pancreatic swelling with significant peripancreatic inflammatory exudation, suggestive of necrotizing pancreatitis, despite consistently normal serum amylase and lipase levels. Treatments including ulinastatin, pantoprazole, and gefarnate provided temporary symptom control. However, symptoms progressively worsened, and nausea and vomiting appeared.

Repeat abdominal contrast-enhanced CT demonstrated pancreatic enlargement, diffuse retroperitoneal soft tissue masses, and involvement extending to multiple adjacent structures. Serum IgG4 was within normal range, and empiric antibiotic treatment provided no benefit. Due to suspicion of retroperitoneal fibrosis or IgG4-related disease, she was referred to our department for further evaluation.

On admission, physical examination revealed stable vital signs, marked weight loss of approximately 10 kg over one month, and evident bilateral lower limb edema (more prominent on the left). Cardiopulmonary examination was normal except for minimal pericardial effusion by echocardiography. Neurological examination was unremarkable.

Admission laboratory tests are summarized in **Table 1**. Of note, she had mild leukopenia (3.72 × 10^9/L), significant anemia (hemoglobin 74 g/L), hypoalbuminemia (24.8 g/L), hyperuricemia (611 μmol/L), and elevated inflammatory markers (ESR 37 mm/h, CRP 71.3 mg/L, IL-6 54.37 pg/mL). Complement C3 level was mildly reduced (0.71 g/L). Autoantibody tests, including antinuclear antibodies, rheumatoid factor, and anti-neutrophil cytoplasmic antibodies, were negative. Notably, anti-endothelial cell antibody was positive at 1:160. Serum and urine immunofixation electrophoresis did not detect monoclonal immunoglobulins, and IgG4 remained within normal limits.

**Table 1:** Comparative analysis of pancreatic involvement in Erdheim-Chester disease.

Case Author /Year,Ref.	Gender/Age	Main Symptoms	Pancreatic Findings	Other System Involvement	Pathology Features	Genetic Mutation	Treatment
Rafiee et al., ​/2023 (6),	Female/73	Abdominal pain, fatigue	Irregular mass in the pancreatic tail, FDG-PET showing hypermetabolic lesions	Bones, cardiovascular, kidneys, pleura	Lipid-laden histiocytes, CD68+, CD1a-	No BRAF mutation	Cladribine
Poehling et al., /1984,​ (7)	Female /57	Lower extremity pain, gait instability	Pancreatic infiltration (discovered at autopsy)	Kidneys, bones	Lipid-laden histiocytes	Not reported	Corticosteroids
Maruyama et al., /2024​ (8),	Male/67	Incidental finding	Diffuse pancreatic enlargement, hyperenhancement on EUS	“Hairy kidney”, bones	CD68+, CD163+, CD1a-, BRAF V600E+	BRAF V600E+	Corticosteroids
Dai et al., /2022​ (9),	Female/29	Diabetes insipidus, lethargy	Hypointense nodules in the pancreatic body and tail, no malignancy on EUS-FNA	CNS, bones	S100+, CD1a-, no BRAF mutation	No mutation	IFN-α
Present Case	Female/58	Recurrent abdominal pain, weight loss	Diffuse pancreatic enlargement, biopsy confirming ECD pathology	Bones, retroperitoneum, kidneys	CD68+, CD163+, BRAF V600E+	BRAF V600E+	Corticosteroids

PET-CT scans showed diffuse hypermetabolic lesions involving pancreas, kidneys, adrenal glands, retroperitoneum, peritoneum, subcutaneous tissues, and bones. Vascular ultrasound identified significant intima-media thickening and stenosis, including subclavian steal phenomenon. Imaging suggested extensive infiltration and multisystem involvement, prompting biopsy.

A CT-guided biopsy of the retroperitoneal and pancreatic tissue showed extensive infiltration by lipid-laden foamy histiocytes positive for CD68 and CD163, negative for CD1α and S-100, and harboring the BRAF V600E mutation. These findings established the diagnosis of Erdheim-Chester disease (ECD). Additionally, characteristic bilateral sclerotic bone changes in femurs and tibias were noted radiographically.

Corticosteroid therapy provided symptomatic relief, but definitive targeted therapy was delayed due to COVID-19 pandemic restrictions. Through telephone follow-up, we later learned she received targeted treatment at another institution after pandemic-related limitations eased, achieving substantial clinical improvement, although detailed follow-up imaging was unavailable.

## Discussion

**Figure 1:**
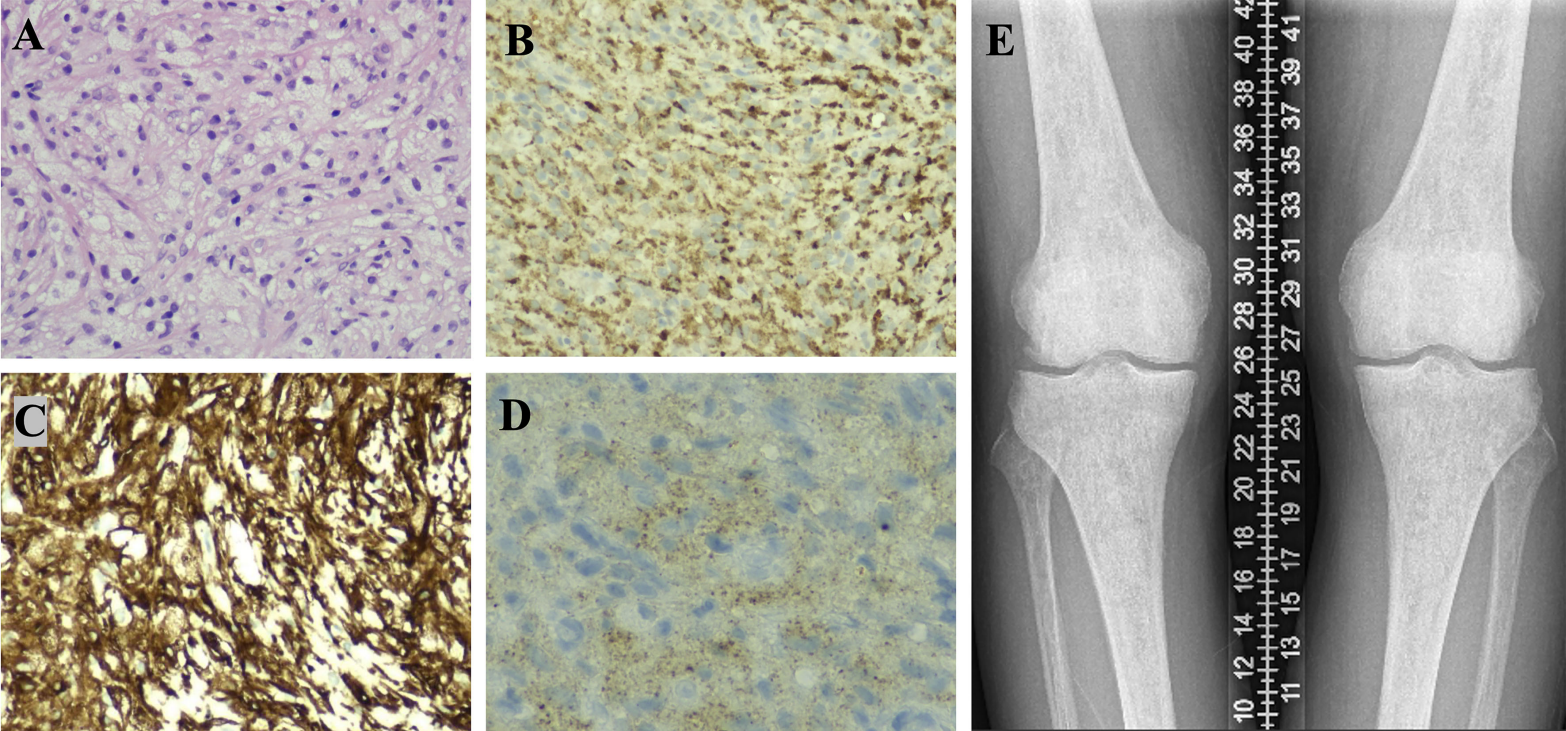
Part 1, Pathological findings from a CT-guided pancreatic biopsy. The pancreatic tissue was destroyed, and the tissue of pathological changes shows diffuse proliferation of spindle-shaped or round cells with abundant, lightly stained cytoplasm, some exhibiting a foamy appearance. The nuclei are mild, with irregular shapes and fine chromatin, and no mitotic figures are observed. The background displays mild lymphocytic infiltration [**(A)**, H&E×200]. Immunohistochemical results indicate IgG focal +, IgG4 -, CD68 +++ [**(B)**, IHC×200], CD163 +++ [**(C)**, IHC×200], CD1α -, S-100 -, and BRAF V600E + [**(D)**, IHC×400, granular intracytoplasmic expression], consistent with a diagnosis of Erdheim-Chester Disease (ECD), characterized by lipid-laden histiocytic proliferation. Part 2. X-ray examination reveals unevenly increased bone density in the distal femurs and proximal tibias of both lower limbs **(E)**.

Erdheim-Chester disease (ECD) is a rare non-Langerhans histiocytosis increasingly recognized as a clonal myeloid disorder driven by mutations in the mitogen-activated protein kinase (MAPK) signaling pathway—most notably the BRAF V600E mutation (11, 12). This mutation, observed in approximately 50–65% of cases, emphasizes the neoplastic nature of ECD and offers significant therapeutic targets (13). However, ECD frequently presents with inflammatory symptoms that mimic infections, malignancies, or autoimmune diseases, complicating early diagnosis (14). Our case exemplifies these challenges, ultimately traced to diffuse pancreatic involvement confirmed through histopathology and molecular testing (**Figure 1**), a rare and diagnostically elusive manifestation (**Table 2**) (6–9).

**Table 2:** Admission laboratory results.

Parameter	Result	Reference Range
White Blood Cells (WBC)	3.72 × 10^9/L	4.0–10.0 × 10^9/L
Hemoglobin (Hb)	74 g/L	115–150 g/L (female)
Mean Corpuscular Volume (MCV)	79.8 fL	80–100 fL
Mean Corpuscular Hemoglobin (MCH)	24.6 pg	27–33 pg
Platelets (PLT)	291 × 10^9/L	100–300 × 10^9/L
Serum Iron	4.1 μmol/L	10.7–30.4 μmol/L
Unsaturated Iron-Binding Capacity (UIBC)	11.5 μmol/L	20–50 μmol/L
Total Iron-Binding Capacity (TIBC)	15.6 μmol/L	50–70 μmol/L
Transferrin	77 mg/dL	200–360 mg/dL
Total Protein	42.9 g/L	60–80 g/L
Albumin	24.8 g/L	35–50 g/L
Prealbumin	72 mg/L	200–400 mg/L
Creatinine	89 μmol/L	50–97 μmol/L (female)
Urea	8 mmol/L	2.9–7.1 mmol/L
Uric Acid	611 μmol/L	150–357 μmol/L (female)
eGFR	61.74 mL/min/1.73m²	> 90 mL/min/1.73m²
Calcium	1.92 mmol/L	2.20–2.55 mmol/L
Triglycerides	1.97 mmol/L	0.29–1.83 mmol/L
LDL-C	1.92 mmol/L	< 3.12 mmol/L
Amylase	56 U/L	35-135U/L
Lipase	48 U/L	8-78U/L
ESR	37 mm/h	0–20 mm/h (female)
CRP	71.3 mg/L	< 8 mg/L
Interleukin-6 (IL-6)	54.37 pg/mL	< 7 pg/mL
Anti-Endothelial Cell Antibody	1:160 Positive	Negative
ANA, ANCA, AMA, SMA, LKM, RF, Coombs’ Test	All Negative	Negative
Serum IgG / IgG4	Normal	Variable normal range
Complement C3	0.71 g/L	0.8–1.6 g/L
Serum/Urine Protein Electrophoresis	No monoclonal band	No monoclonal band
Urine Kappa Light Chain	10.7 mg/L	Slightly elevated
Urine Lambda Light Chain	5.56 mg/L	Slightly elevated
Procalcitonin (PCT)	0.557 ng/mL	< 0.05–0.1 ng/mL
T-SPOT.TB	Negative	Negative
Tumor Markers	Normal	Within normal ranges

In our patient, prolonged symptoms of recurrent abdominal pain, retroperitoneal soft tissue thickening, and hydronephrosis initially suggested differential diagnoses including tuberculosis, obstructive uropathy, or autoimmune pancreatitis. Notably, the patient’s normal serum IgG4 levels were instrumental in excluding IgG4-related disease, another condition known for forming mass-like pancreatic lesions. Nonetheless, clinicians should remain aware that IgG4-negative cases exist and may complicate the diagnostic picture (15, 16). The histopathological presence of foamy histiocytes expressing CD68 and CD163, without CD1α or S-100 positivity, and detection of the BRAF V600E mutation confirmed ECD diagnosis in this patient. These findings underscore the importance of molecular diagnostics for definitive identification and targeted therapy planning (3).

Long-bone involvement, characteristic of ECD, is often asymptomatic and detectable by imaging rather than clinical presentation, highlighting the diagnostic utility of skeletal surveys (17). Our patient exhibited typical bilateral sclerotic lesions in distal femurs and proximal tibias, reinforcing this diagnostic hallmark.

From an immunopathological standpoint, ECD occupies an intersection between myeloid neoplasms and chronic inflammatory conditions (18). The pathological histiocytes in ECD originate from activated monocyte/macrophage lineages, displaying markers consistent with M2-polarized macrophages (CD163-positive), producing pro-inflammatory cytokines such as IL-1, IL-6, and TNF-α. These cytokines exacerbate local tissue damage, promote fibrosis, and recruit additional immune cells, perpetuating inflammation and multi-organ dysfunction. Consequently, therapeutic approaches targeting these signaling pathways can significantly alter disease progression and patient outcomes.

Traditional treatments for ECD, including corticosteroids, interferon-α, and cytotoxic chemotherapy, provide variable symptom relief but rarely sustained remission. The recent introduction of targeted therapies such as BRAF and MEK inhibitors markedly changed the treatment landscape, offering profound clinical improvements for patients harboring the BRAF V600E mutation (10). However, real-world challenges—highlighted by the delayed initiation of targeted therapy in our patient due to the COVID-19 pandemic—illustrate existing barriers in accessing advanced treatment options promptly.

Our patient initially experienced symptomatic relief with corticosteroids, consistent with transient immunosuppressive benefits, but achieved substantial clinical improvement only after subsequent targeted therapy administered at another institution. Unfortunately, detailed follow-up imaging studies post-treatment were unavailable due to external circumstances, limiting comprehensive evaluation of treatment efficacy—a recognized limitation of this report.

**Figure 2:**
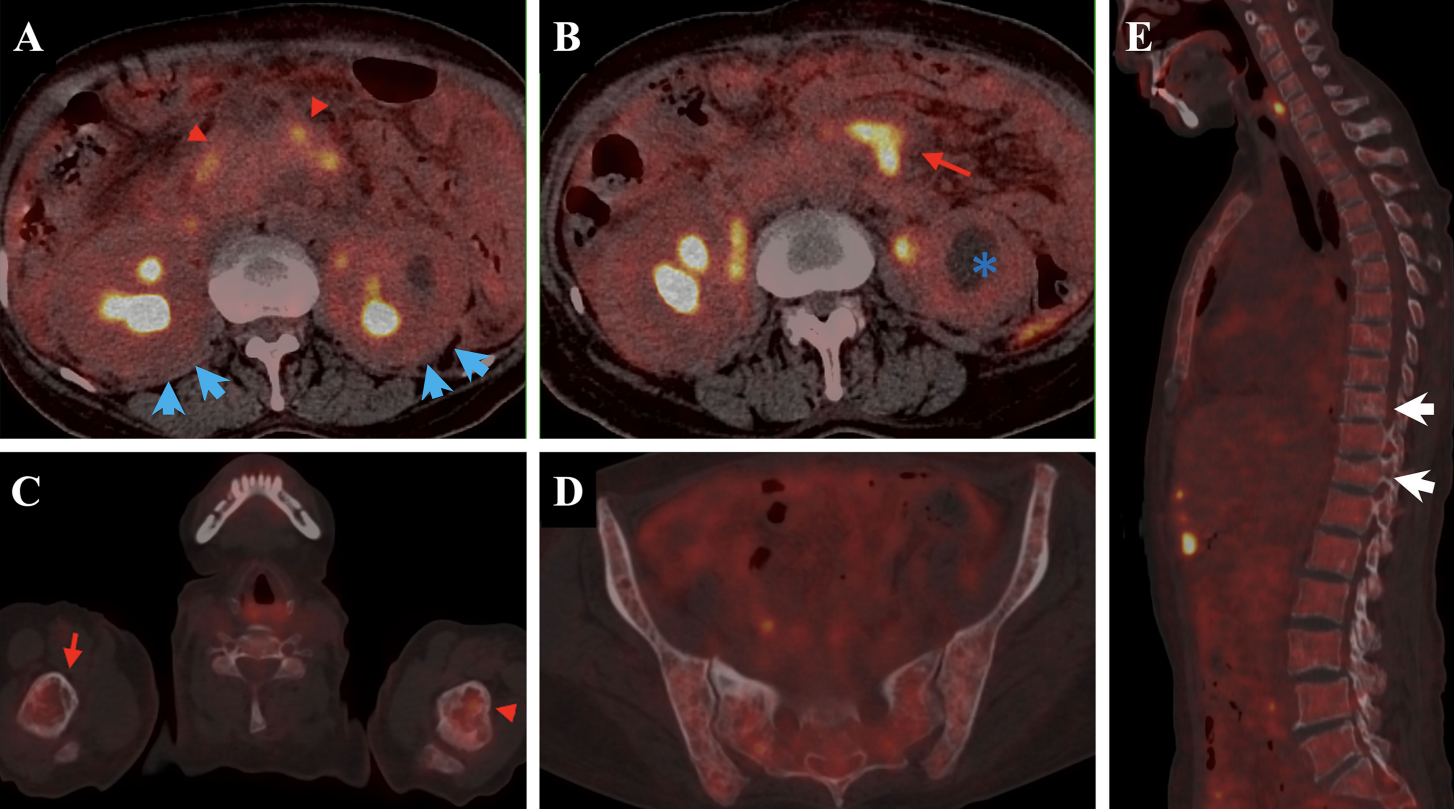
The patient’s PET-CT demonstrates involvement of multiple tissues and organs. Notably, pancreatic abnormalities [**(A)**, red triangle] and bilateral renal enlargement with hydronephrosis [**(B)**, blue asterisk] are observed, along with mild “hairy kidney” appearance [**(A)**, blue arrow]. There are soft tissue density shadows adjacent to the abdominal aorta in the retroperitoneum and bilateral iliac vessels [**(B)**, red arrow], all exhibiting increased glucose uptake. Additionally, uneven bone density is observed in the spine and pelvic bones, with multiple patchy low-density areas **(C–E)**. Compression fractures are noted at the T9 and T11 vertebral.

Additionally, our case demonstrated multisystem involvement, notably including rare pancreatic and renal manifestations. Pancreatic involvement in ECD is exceedingly uncommon, previously described in only a few reports, and easily misinterpreted as autoimmune pancreatitis or malignancy (19). Therefore, clinicians must maintain a high index of suspicion for ECD when encountering atypical pancreatic lesions coupled with systemic involvement, particularly with characteristic imaging findings such as hypermetabolic lesions on PET-CT scans and skeletal abnormalities (**Figure 2**) (20).

This case further underscores the complex interplay between inflammation and oncogenesis within ECD, highlighting its immunological and neoplastic dimensions. Future research into cellular interactions and signaling pathways driving ECD pathogenesis may provide broader insights into similar inflammatory and neoplastic disorders, potentially guiding innovative therapeutic strategies.

In summary, our report contributes to the limited data on pancreatic ECD, emphasizing the critical importance of integrating clinical presentations, imaging, histopathological findings, and molecular diagnostics for accurate diagnosis. Timely identification of ECD, particularly with unusual organ involvement, facilitates early initiation of targeted therapies, markedly improving patient prognosis and quality of life. For clinicians and researchers focusing on immune-mediated disorders, ECD exemplifies the delicate intersection of chronic inflammation and malignancy, warranting continued exploration.

## Data Availability

The original contributions presented in the study are included in the article/supplementary material. Further inquiries can be directed to the corresponding author.
